# Monitoring Poisson’s Ratio Degradation of FRP Composites under Fatigue Loading Using Biaxially Embedded FBG Sensors

**DOI:** 10.3390/ma9090781

**Published:** 2016-09-19

**Authors:** Erdem Akay, Cagatay Yilmaz, Esat S. Kocaman, Halit S. Turkmen, Mehmet Yildiz

**Affiliations:** 1 Faculty of Aeronautics and Astronautics, Ayazaga Campus, Istanbul Technical University, Maslak, Istanbul 34469, Turkey; erdemakay@itu.edu.tr (E.A.); halit@itu.edu.tr (H.S.T.); 2 Faculty of Engineering and Natural Sciences, SU-Kordsa Composite Technologies Center of Excellence and Integrated Manufacturing Technologies Research and Application Center, Sabanci University, Orhanli-Tuzla, Istanbul 34956, Turkey; cagatayyilmaz@sabanciuniv.edu (C.Y.); esatselim@sabanciuniv.edu (E.S.K.)

**Keywords:** strain, Poisson’s ratio, FBG, composite, fatigue, lateral strain

## Abstract

The significance of strain measurement is obvious for the analysis of Fiber-Reinforced Polymer (FRP) composites. Conventional strain measurement methods are sufficient for static testing in general. Nevertheless, if the requirements exceed the capabilities of these conventional methods, more sophisticated techniques are necessary to obtain strain data. Fiber Bragg Grating (FBG) sensors have many advantages for strain measurement over conventional ones. Thus, the present paper suggests a novel method for biaxial strain measurement using embedded FBG sensors during the fatigue testing of FRP composites. Poisson’s ratio and its reduction were monitored for each cyclic loading by using embedded FBG sensors for a given specimen and correlated with the fatigue stages determined based on the variations of the applied fatigue loading and temperature due to the autogenous heating to predict an oncoming failure of the continuous fiber-reinforced epoxy matrix composite specimens under fatigue loading. The results show that FBG sensor technology has a remarkable potential for monitoring the evolution of Poisson’s ratio on a cycle-by-cycle basis, which can reliably be used towards tracking the fatigue stages of composite for structural health monitoring purposes.

## 1. Introduction

Composites, such as glass or carbon fiber-reinforced polymer structures, have received significant attention as structural materials due to their high specific strength and stiffness. On the other hand, when compared with metals, composite structures suffer from the lack of a well-characterized fatigue life and damage mechanisms. In order to assess the structural reliability of the components during their service life, it is essential to detect the damage formation.

Strain is one of the most important measurable parameters for lab-scale testing and monitoring the in-service behavior of composite materials under applied loads. Strain-gauges or strain-gauge-based extensometer sensors have been commonly used to collect strain data for a long time. Due to their working principle based on measuring the change in the electric resistance of the metallic foil of the gauge, strain-gauge sensors are very sensitive to electromagnetic fields and, hence, require the usage of appropriate filters to eliminate unwanted external noises. On the other hand, because of their size, standard strain-gauges are not suitable for being embedded into structural elements. Moreover, they have to be attached to an object by using adhesives, such as cyanoacrylate, after careful surface treatment. Thus, surface-mounted strain-gauges do have very low fatigue performance and are not suitable for high cycle and high amplitude fatigue conditions. Due to the above-mentioned reasons, different sensor technologies have been investigated for years to circumvent the limitations of the strain-gauge technology. The studies conducted during the last decades show that the Fiber Bragg Grating (FBG) sensors are very effective for measuring various parameters (i.e., temperature, pressure, strain, etc.) [1,2]. An FBG sensor is an optical filter that can be written on the core of a single mode optical fiber at various gauge lengths through periodically changing the refractive index of the core along the length [3]. Upon being exposed to a broad band of light sent through the optical fiber, the FBG sensor refracts a narrow light signal with a specific center wavelength (i.e., $\lambda_{B}$, Bragg wavelength) back to the light source while allowing the remaining portion of the light to pass. The center wavelength of the reflected spectrum is a function of the grating pitch (i.e., Λ, periodic variation of the refractive index) and the effective refractive index (i.e., $n_{eff}$), as given in Equation (1):

(1) $$\lambda_{B}=2n_{eff}\Lambda .$$

The Bragg wavelength shifts if a mechanical or thermal load changes the spacing between the periodic refractive index of the sensor region. The amount of the wavelength shift can be easily transformed into strain or temperature data by using the expression below:

(2) $$\frac{\Delta \lambda }{\lambda_{B}}=\left(1-p_{e}\right)\varepsilon +\left(\alpha +\xi \right)\Delta T,$$

where *ε* and ΔT are the axial strain and temperature change of the grating region, respectively. $p_{e}$, *α* and *ξ* are the effective photo-elastic constant, thermal expansion coefficient and thermo-optic coefficient, respectively. Being small and flexible, FBG sensors can be embedded discretely into composites at locations of interest without endangering the structural integrity of the host material. This makes them very convenient for monitoring the internal behavior of composites through acquiring local strain distribution and evolution, which can be used for damage analysis of structures [4,5]. Strain measurement with embedded FBG sensors becomes particularly important to monitor the long-term behavior of structures as in the case of fatigue loading, since surface-mounted strain-gauges do have very low fatigue resistance and are not suitable for high cycle and/or high strain amplitude fatigue applications. In the literature, it was proven that FBGs embedded into FRP (Fiber-Reinforced Polymer) composite structures could still be intact and operative after being subjected to millions of cyclic loadings without any sign of deformation and degradation [6]. Thus, the fatigue resistance of FBG sensors makes them perfect strain measurement tools for the long period of cyclic loads.

Several different methods have been proposed and investigated to study the accumulation of damage and the damage state of composite structures under static and dynamic loading conditions. The reduction of the elastic modulus was investigated as a damage indicator for composite materials [7]. The remaining useful life prediction model was proposed and implemented on glass fiber-reinforced composite structures, which was based on cycle by cycle expended energy calculation wherein FBG-measured strain was an input parameter [6]. Several research works proposed to use the reduction in Poisson’s ratio as the sensitive damage parameter [8,9,10,11] that may reveal the damage state of composite materials subjected to dynamic and static loading conditions, since Poisson’s ratio is influenced by the transverse crack density. Smith and Wood used shear-lag analysis to model the change in Poisson’s ratio under the static loading condition and compared the analytical results with experiments. They used rosette-type strain-gauges and observed that the reduction of Poisson’s ratio was related to the transverse crack density [12]. Gao et al. monitored damage accumulation in carbon fiber composites and indicated that the reduction in Poisson’s ratio was much greater than the reduction in the modulus of elasticity for the applied axial strains by using strain-gauges in their experiments [8]. Surgeon et al. also performed some experiments by using rosette-type strain-gauges on prepreg carbon fiber composites and verified that the damaged state of materials had a significant amount of reduction in Poisson’s ratio compared to undamaged ones [9]. Paepegem et al. investigated the behavior of Poisson’s ratio $\nu_{xy}$ with respect to applied longitudinal strain $\varepsilon_{xx}$ and observed a repeatable correlation between $\nu_{xy}$ and $\varepsilon_{xx}$ by measuring these parameters on the specimen surface using strain-gauges and optical sensors [10].

In all of the above studies, Poisson’s ratio monitoring of composite materials under either dynamic and static loading conditions was performed mainly using surface-mounted strain-gauges, optical sensors or extensometers, which provide strain data obtained from the material surface. Reliable measurement of axial and lateral strain is vital for Poisson’s ratio monitoring. Poisson’s ratio monitoring using strain acquired from the surface of orthotropic FRP composite structures might be rather erroneous given that the behavior of outer lamina might be significantly different from the inner structure. In real applications, it is important and critical to track the variation in Poisson’s ratio with respect to fatigue life or the number of cycles of the composite, whereby the fatigue life and the damage state of the composite can be monitored more effectively and conveniently. Therefore, in this study, we have monitored the evolution of Poisson’s ratio of the composite materials subjected to fatigue loading as a function of fatigue cycle number using embedded axial and transverse FBG sensors. To our best knowledge, the evolution of Poisson’s ratio of glass fiber-reinforced epoxy composites using embedded biaxial FBG sensors has not been presented in the open literature yet. Moreover, we correlated the variation of axial force and temperature on the composite during fatigue with the evolution of Poisson’s ratio, thereby showing that Poisson’s ratio measured by embedded FBG sensors is a reliable quantity, which can be used as a damage index.

## 2. Methods

### 2.1. Test Specimens and Sensor Placement

Glass Fiber-Reinforced Polymer (GFRP) composite specimens are manufactured by using an RTM (Resin Transfer Molding) method, which is a closed mold technique with inlet and outlet ports for resin flow. In this method, the resin system is injected into a mold cavity (where layers of dry fiber reinforcement are placed) under constant pressure or flow rate using either a pressurized container or resin injection system. The injected resin impregnates dry fabrics, and then, excess resin is discharged off the outlet port into a vacuumed catch-pot. RTM systems employ different types of heating subsystems integrated into the mold to cure the impregnated fabric, namely water or electric heaters, wherein this study, the RTM system with water heating capability is used. RTM systems generally deliver high quality and industry standard FRP composites with high a fiber volume fraction.

**Figure 1:**
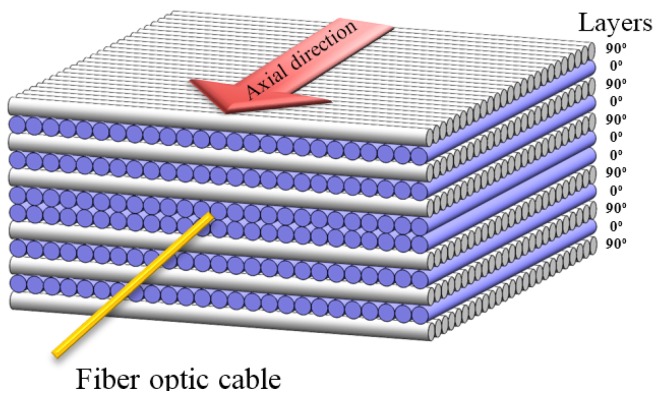
Schematic of the laminate configuration with an embedded FBG sensor.

**Figure 2:**
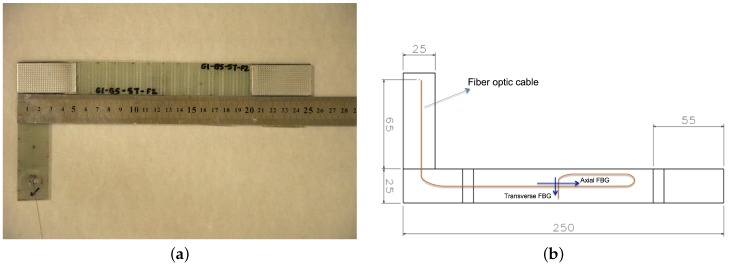
L-shaped fatigue test specimen (**a**) and FBG sensor placement within the specimen (**b**).

As a reinforcement material, 313 gsm biaxial (0/90) E-glass stitched fabric (Metyx LT300 E10A) with 161 gsm along the warp (0°) direction (which is the loading direction of the specimen) and 142 gsm along the weft (90°) direction (which is perpendicular to the loading direction) was used. As a matrix material, Araldite LY 564 epoxy and the XB3403 hardener system purchased from Huntsman were utilized. The RTM system employed for manufacturing GFRP composite plates has a special design that enables the embedding of fiber optic sensors into the composite plate during fabrication [12,13]. Resin was injected into the mold by applying external pressure up to 2 bars. Composite samples underwent an initial cure process at 65 °C for 24 h and then a post cure for another 24 h at 80 °C. The layer configuration of the GFRP plates is [90/0]_6s_, and is shown in Figure 1. The fiber volume fraction of composite plates manufactured by the RTM method was determined to be around 50%. The composite panels were cut into mechanical test specimens using a water-cooled diamond circular blade saw, such that they have final dimensions of 250 mm × 25 mm × 3.7 mm with a 150-mm gauge length. The ingress/egress of FBG sensors into composite structures is a challenge and becomes particularly difficult in closed mold systems, such as RTM, unlike other composite manufacturing methods, namely vacuum infusion and hand lay-up, where FBG sensors can ingress and egress composite structures at the edges. Nevertheless, in closed mold systems, the FBG sensors need to egress composite parts from the surface [12,13]. As for the mechanical test specimens with embedded FBG sensors, the egress should be outside the gauge length of the specimen, not to endanger the mechanical performance of the specimen. To be able to clamp the test specimen with the standard grip heads of the fatigue system without the need for custom-made gripping fixtures, the section of the composite plate including an FBG sensor was cut into an L-shape, as shown in Figure 2a, so that the specimen can be easily clamped by the grips of the fatigue test platform without damaging the egress region of the FBG sensors. In this study, we produced three composite plates with FBG sensors (i.e., Specimen 1 from the first plate, Specimen 2 from the second plate and Specimen 3 from the third plate), which were processed into several test specimens with and without the FBG sensor for static and fatigue testing.

Two one millimeter-long FBG sensors with a distance of 21 cm (purchased from Technica SA) were embedded into the center of the each specimen’s gauge area within the plane of symmetry of the laminate (i.e., between Layers 6 and 7). The first FBG sensor is positioned along the loading direction (i.e., the axial direction), while the second one is oriented along the transverse direction (i.e., lateral direction), so that both sensors are perpendicular to each other on the same fiber optic cable, as seen in Figure 2b. The pre-calculated distance between the two FBG sensors (i.e., 21 cm) provided the largest possible bend radius during the placement of the fiber optic cable to minimize the optical losses. Axial and transverse FBG sensors have the center wavelengths of 1550 nm and 1540 nm, respectively. To ensure that the FBG sensors preserve their initial positions during the handling and manufacturing stages, fiber optic cable was fixed onto 0° ply (i.e., the 6th layer) by passing it through the fabric stitches prior to the manufacturing.

### 2.2. Fatigue Testing

The MTS Universal Testing Machine (UTM) with an MTS 322 test frame, MTS 647 hydraulic wedge grips, MTS FlexTest GT digital controller and MTS Station Manager software was used for all of the mechanical tests in this study. Load, displacement and strain data were collected with a built-in load cell, Linear Variable Differential Transformer (LVDT) and an MTS 634.25F-24 model axial extensometer, respectively. In addition, temperature and strain data were collected from the surface of specimens with a K-type thermocouple and biaxial strain-gauge, respectively, using a National Instruments NI SCXI-1000 data acquisition system with Signal Express software at a 100-Hz sampling rate. The wavelength data of FBG sensors were acquired by a Micron Optics SM130-700 interrogator using Micron Optics Enlight software at a sampling rate of 100 Hz. Fatigue tests were performed at 4 Hz of cyclic load frequency to circumvent the excessive autogenous heating of fatigue specimens.

## 3. Results and Discussion

**Figure 3:**
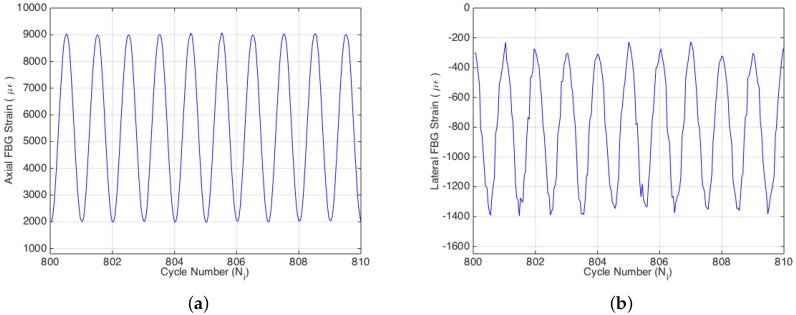
Ten consecutive strain cycles for axial (**a**) and lateral (**b**) FBG sensors.

To understand the effect of the control type of the fatigue experiment on the fatigue behavior of specimens, as well as on the FBG sensor, two different experimental procedures were applied, such that in first and third experiments, the strain amplitude was kept constant during the cyclic loading through using an extensometer as a control sensor, while in the second one, the displacement amplitude was retained as constant by employing LVDT as a control sensor. During the experiments, the peak and valley of axial strain, displacement and force for each sinusoidal fatigue cycle were acquired by the MTS system. Meanwhile, axial and lateral strains of the mid-plane of the composite specimens were also obtained by FBG sensors. Temperature compensation is applied (i.e., approximately 25 με/°C) to the entire FBG data of all three experiments using the thermocouple data. In Figure 3 is given the sinusoidal variation of the strain acquired by both the axial and transversal FBG sensors during the fatigue experiment. Poisson’s ratios of the specimens were calculated for each cycle using biaxial strain data obtained by the FBG sensors. The reduction of Poisson’s ratio with respect to the cycle number was monitored, which is an important parameter for the evaluation of the fatigue damage.

### 3.1. Strain Controlled Fatigue Test

Fatigue in fiber-reinforced polymer matrix composites is characterized by three distinct phases. Namely, in the first phase, comprising the first 10%–20% of fatigue life, the rapid formation and interconnection of matrix cracking due to residual curing stresses and discontinuities within the composite causes a sharp, non-linear decrease in stiffness. The second phase corresponds to between 10%–20% and 90%–95% of the fatigue life, where there is a gradual and linear decrease in stiffness, which is attributed to matrix crack propagations, fiber debonding and delamination. The final phase is characterized by a sharp nonlinear decrease in stiffness due to the plurality of fiber breakages [6].

**Figure 4:**
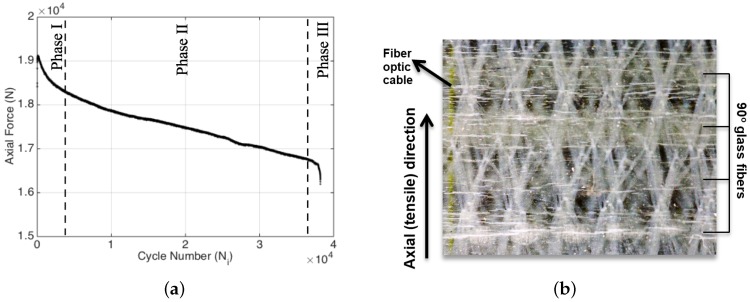
Force versus cycle number variation (**a**) and the transverse matrix cracks after 38,000 load cycles (**b**) for Specimen 1.

Specimen 1 was subjected to an extensometer-controlled tension-tension constant strain amplitude fatigue at a strain ratio (i.e., test strain/ultimate strain) of 0.55. Figure 4a indicates that the evolution of the applied fatigue force as a function of cycle number also possesses three distinct fatigue phases as expected, since the stiffness reduction and decrease in the applied force are equivalently related one another for the fatigue experiment conducted at a constant strain amplitude, as is the case for the current study. In this experiment, the first fatigue phase spans over about 4000 cycles (corresponding to nearly 10% of the failure cycle) in which the force on the test specimen continuously decreases as expected since the fatigue experiment is performed under constant strain, and as a result of fatigue damage, in each cycle, less and less force is required to reach the initially preset strain value. Recall that the cause of such a noticeably sharp decline has been associated with the matrix cracking, where in this particular loading configuration, the damage is likely to be dominated by transverse cracks. After 4000 cycles, the descent in the force continues, albeit with a smaller rate compared to the first region, and almost follows a linear trend up to the last 2000 cycles where this phase is referred to as the second phase of the fatigue. Then, rather close to the failure, which generally corresponds to 5%–10% of the overall fatigue life, the third phase is entered, which is characterized by a drastic drop in the applied force.

Previous studies stated that matrix cracks were one of the most dominant damage mechanisms for FRP composites [14,15,16,17]. Transverse matrix cracks take place in 90° plies and cause degradation in material properties as fatigue cycles continue. In Figure 4b, numerous transverse cracks within the specimen are shown in a high-resolution camera image with a black background. Owing to the transparent characteristic of the glass fiber/epoxy composite, the embedded fiber optic cable can be seen in yellow color on the very left of the figure. On the right side of the figure, tows of 90° glass fibers are indicated. The stitches of the biaxial fabric are also visible as wavy white lines. The transverse matrix cracks perpendicular to the tensile direction are visibly spread out across the entire surface.

**Figure 5:**
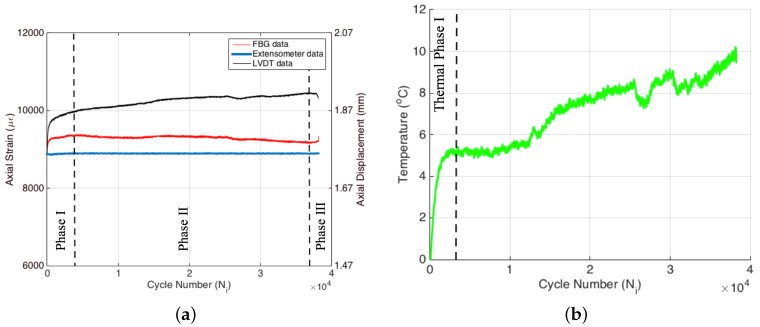
Extensometer, FBG and Linear Variable Differential Transformer (LVDT) data (**a**) and temperature variation (**b**) for Specimen 1.

Figure 5a shows the evolution of maximum strain (i.e., peak strains in the sinusoidal strain form) and displacement as a function of cycle number, which were recorded by extensometer, FBG and LVDT sensors where the left axis belongs to the extensometer and the FBG sensor, while the right axis is for LVDT. Since the test was performed under extensometer control, the gauge length within the area of extensometer blades was exposed to a peak strain of 8900 με (i.e., corresponding to the strain ratio of 0.55) for each cyclic load. The strain data of the extensometer in Figure 5a are perfectly flat throughout the test as expected. The failure of Specimen 1 took place around 38,000 cycles.

**Figure 6:**
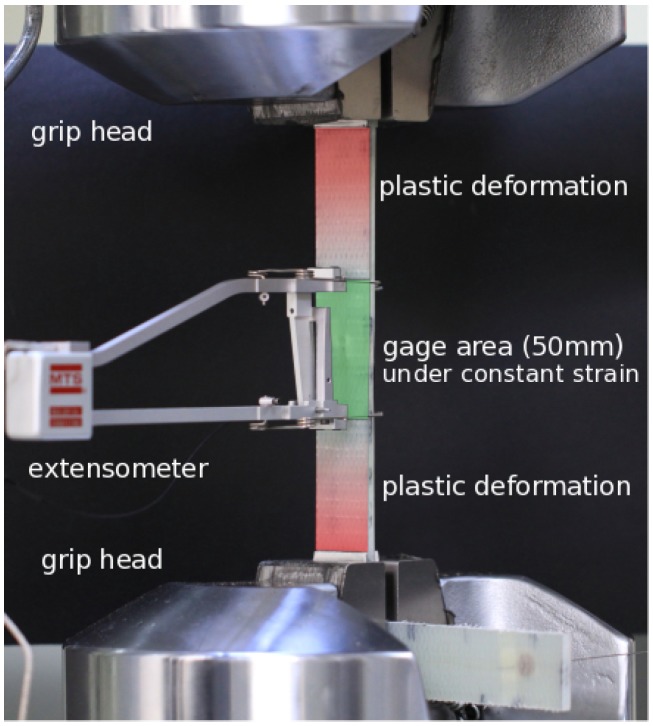
Deformations during an extensometer-controlled fatigue experiment.

Displacement data collected by LVDT yield a different behavior when compared to the strain data of the extensometer, such that the overall applied displacement gradually increases during the test, as seen in the right axis of Figure 5a, even though the applied strain is constant in the extensometer gauge-length. This phenomenon, thoroughly investigated in another study [18], is essentially caused by the cracks and damage accumulated within the entire length of the specimen. During the extensometer-controlled fatigue experiment, the gauge area (i.e., green area in Figure 6) is subjected to constant strain amplitude, thereby leading to constant average displacement within the gauge length of the extensometer. On the other hand, due to the permanent deformation out of the gauge area of the specimen, the grip head of the universal testing machine should move further to impose a predefined strain level on the gauge length of the extensometer. The crack density of the specimen directly affects the progression of this permanent deformation. Thus, the permanent deformation results in an increased displacement at each cyclic load. The small amount of fluctuations in the slope of the displacement curve is most probably related to the transverse matrix cracks, delamination and fiber breakages induced by fatigue loading at particular periods of fatigue life. Interestingly, there are three distinct phases in the variation of displacement during the entire fatigue test, namely the sharp and non-linear increase in the displacement followed by the nearly linear increase and final sudden drop. These phases are well correlated with the force versus cycle number plot presented in Figure 4a.

It should be noted that the raw FBG data are in the form of wavelength and therefore need to be calibrated with respect to sensors whose outputs are strain (i.e., extensometer and strain-gauges). To this end, before fatigue tests, specimens with FBG sensors were loaded in a quasi-static cyclic manner four times until reaching the desired strain ratio to calibrate the FBG sensor, as well as eliminate any slippage of the sample from the grips during fatigue testing. FBG sensors were calibrated with respect to both the extensometer and strain-gauge using the data in the fourth loading cycle. The FBG calibration coefficients calculated based on the extensometer and strain-gauge data might be slightly different due to the dissimilar gauge length between the strain-gauge and extensometer. In this study, the extensometer-based calibration coefficient is utilized, namely 1.25 pm/με. Recalling that the fatigue test on this specimen was conducted under constant strain using the extensometer, one may at first sight expect that FBG sensors should also give constant strain values since they are flanked by the two pins of the extensometer. However, maximum strains gathered from the FBGs can be significantly distinct from the global strain of the specimen. As the fatigue experiment progresses, the local strains measured by the FBG sensor vary such that the trend has three different regions, that is to say, an initial increase superseded by a gradual decline, followed by a final sharp rise after which failure occurs. These three phases are in agreement with the fatigue phases observed in both the force and LVDT plots as a function of cycle number in Figure 4a and Figure 5a, respectively. This finding indicates that by means of internal and local strain monitoring with discrete embedded FBG sensors, one can conclude the fatigue phases of the composite specimens.

The drop in the FBG recorded strain during Phase II (i.e., about 200 με), albeit with the increase in the displacement, is related to transverse cracks and damages formed within the specimen. FBG sensors are located at the mid-point of the specimen gauge area (see Figure 2b), which is under constant strain (in the gauge length of the extensometer) during the entire test. The applied strain is an average value over the gauge length of the extensometer (i.e., the green area in Figure 6). On the other hand, FBG sensors are only 1 mm long and record the strain data at the mid-point of the gauge length within the symmetry axis of the specimen. Even though the entire 50 mm of the gauge length is under constant strain at each cyclic load, there is still a considerable amount of damage inside this area that causes a non-uniform strain distribution. Thus, the FBG sensor at the center of the extensometer gauge length experiences less and less strain with respect to the FBG recorded strain at the beginning of Phase II as the damage increases [18].

It is well documented in the literature that when a specimen is subjected to cyclic loading, a portion of the mechanical energy is dissipated as heat (also referred to as autogenous heating), causing a rise in the temperature of the specimen [19,20,21]. For metals and fiber-reinforced composites that are exposed to cyclic loads, the variation of temperature as a function of cycle number (thermal curve) can undergo three different thermal phases, namely an initial, rapid increase in the first phase, followed by a linear change in the second phase and a final non-linear increase near the failure in the third phase [17,19,21]. Such a heat generation in response to the fatigue loading can be the result of several different mechanisms as substantiated in the literature, namely heating owing to viscoelasticity/material damping, heating due to the plastic deformation at the end of the crack tips that are formed due to the repeated loading and, finally, the friction between the internal surfaces of the cracks and deformations [22,23]. Performing a simple energy balance calculation, one can show that the frictional heat generation is a dominating factor in the autogenous heating. The effective thermal conductivity of the composite specimen decreases with the increase in damage density since the discontinuities (cracks and damages) in the composite act as insulating media. Expectedly, the heat generation rate due to damage accumulation or crack formation should be higher than the heat removal rate by the ambient environment in accordance with the k∂T/∂n=hΔT where $\Delta T=T_{s}-T_{\infty }$ and $T_{s}$ and $T_{\infty }$ are the temperatures of the specimen’s surface and the ambient environment; *k* is the thermal conductivity; *h* is the heat transfer coefficient; and ∂/∂n is the spatial derivative along the normal direction. As one can immediately conclude, the smaller the crack density, the smaller the heat generation and the larger the thermal conductivity and the conductive heat flux ($q_{c}=k\partial T/\partial n$) to the boundary that can be removed from the surface through convection ($q_{\mathrm{cv}}=h\Delta T$). As ΔT increases, convective heat flux in the direction perpendicular to the surface of the specimen augments. In Figure 5b, the variation of the temperature of the specimen as a function of fatigue cycle is provided for the entire test, where the temperature data correspond to the difference between the actual temperature (i.e., the temperature during the fatigue cycle) and the initial temperature (i.e., the stabilized temperature before the experiment) of the specimen surface. It can be clearly seen that the temperature of the specimen under fatigue loading increases by around 5 °C in a rather short cycle number, which is roughly 10%–15% of the total cycle number for failure. The length of this first thermal phase is in agreement with that of the first fatigue phase. Recall that in the first fatigue phase, the number of transverse matrix cracks increases with the fatigue cycles. Hence, newly-generated internal surfaces due to the damages are to be exposed to the mechanical load of the fatigue experiment at each cycle. In addition to heat generation because of viscoelastic damping and plasticity, the friction between these newly-generated internal surfaces significantly contributes to the rapid increase in temperature of the specimen during the first fatigue phase. As the experiment continues, the temperature keeps rising, albeit at a smaller rate than the first phase, since the rise in ΔT increases convective heat flux from the surface of the specimen. The nearly linear rise in temperature of the specimen at the second thermal phase indicates that the deformations and damages formed in the course of the second fatigue phase are able to generate heat due to the above-stated mechanisms at a rate higher than the heat removal rate to the surroundings through convection. This kind of behavior in temperature data reveals that the overall damage progressively grows during the fatigue experiment [12]. It is worthy to note that the temperature regimes we observe in our study correlate well with the first, second and third fatigue phases in Figure 4a and Figure 5a.

**Figure 7:**
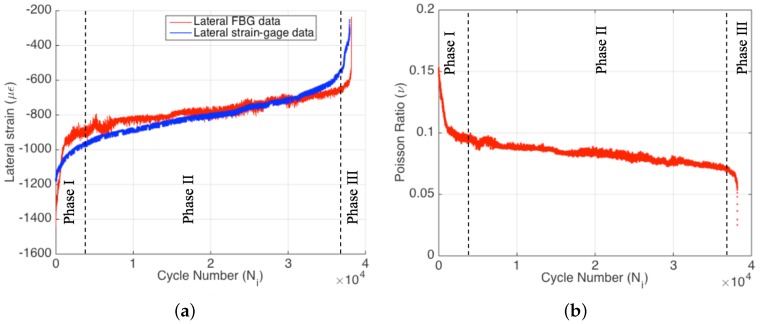
Strain data obtained by the lateral part of the strain-gauge and lateral FBG (**a**) and Poisson’s ratio reduction (**b**) for Specimen 1.

The axial part of the biaxial strain-gauge attached to the specimen surface failed at the very beginning of the experiment (i.e., around 180 cycles). This is directly caused by the high cyclic strain applied to the specimen, which clearly states that conventional strain-gauges are not suitable for such applications. On the other hand, the lateral part of the axial strain-gauge survived until the end of the experiment, since the absolute lateral strain decreased from the 1200–1400 με interval to lower absolute values, as seen in Figure 7a. The survival of the lateral part of the strain-gauge during the entire test also confirms that the strain-gauge attachment to the surface was good enough and that the failure of the axial part of the strain-gauge is not related to any kind of experimental error. One can see from Figure 7a that the FBG and strain-gauge strains are in agreement with each other during the entire test. However, there is a slight difference in the slope between the two dataset. Additionally, the FBG data have more fluctuations in comparison to the strain-gauge data, since the interior of the composite is more sensitive to the crack formations during the fatigue loading. Strain-gauge data are more like a smoothened version of the FBG data, since the surface is possibly less sensitive to the damage and degradation within the specimen. This result indicates that the internally-measured strain differs from the strain acquired on the surface, and the interior sensors are more sensitive to fatigue damage in composites.

It is obvious that the damage accumulation in the course of the fatigue test has a significant effect on the measured parameters, such as force, displacement, axial FBG strain and temperature. Poisson’s ratio is an important engineering constant, and its variation with respect to cycle number can reveal further information on the fatigue behavior of composite structures. Figure 7b presents the variation of the FBG sensor-based Poisson’s ratio $\nu_{xy,i}=-\varepsilon_{y,i}^{FBG}/\varepsilon_{x,i}^{FBG}$ as a function of cycle number for Specimen 1 where $\varepsilon_{y,i}^{FBG}$, $\varepsilon_{x,i}^{FBG}$ and $\nu_{xy,i}$ are transverse and axial strains and Poisson’s ratio at cycle i, respectively. Figure 7b shows that the FBG-based Poisson’s ratio of the composite specimen significantly decreases throughout the test. The cycle-by-cycle basis monitoring of the Poisson’s ratio is well suited to construct the three phases of the fatigue process. Being similar to the other fatigue parameters, Poisson’s ratio also indicates a rapid and nonlinear variation in the first phase of the fatigue test, which is followed by a linear variation characteristic of the second fatigue phase and the final drastic variation corresponding to the third phase of the fatigue. It is clear that biaxially-located FBG sensors can be properly used for fatigue monitoring even for high strain ratios. This gives new opportunities for structural health monitoring systems and fatigue life prediction, since the simultaneous monitoring of the Poisson’s ratio reduction can be a useful tool for this purpose.

### 3.2. Displacement-Controlled Fatigue Test

**Figure 8:**
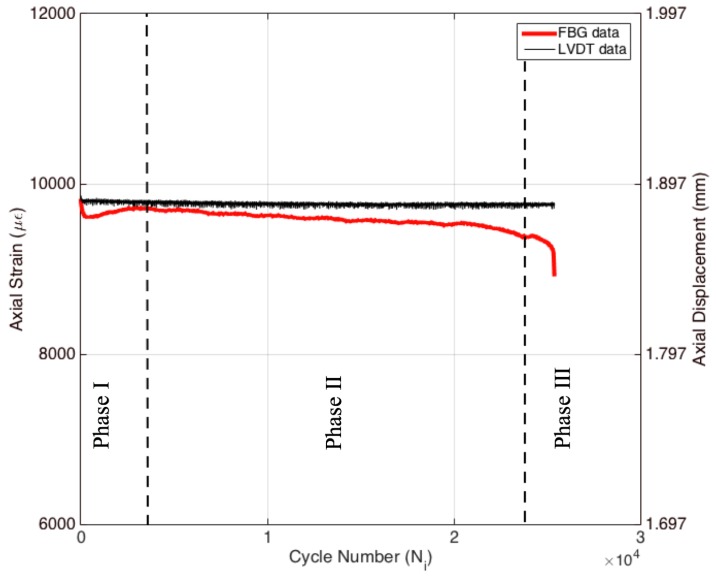
Axial strain data of the FBG and LVDT (left axis) and axial displacement (right axis) for Specimen 2.

**Figure 9:**
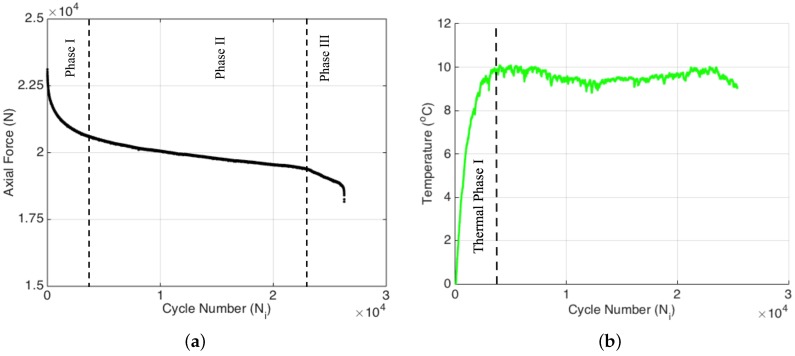
Force data (**a**) and temperature change (**b**) for Specimen 2.

Specimen 2 was subjected to displacement-controlled tension-tension strain cycles during the entire test. The strain ratio (i.e., applied strain/ultimate strain) of 0.6 was applied during the experiment. The displacement data obtained by the LVDT and the strain data acquired by the axial FBG sensor (i.e., calibrated with extensometer) with respect to cycle number are shown in Figure 8. For comparison, LVDT data in the figure were converted from displacement to strain. Since the LVDT system controls the movement of the grip heads, the overall applied displacement is constant throughout this experiment. Thus, the resultant strain values converted from the displacement data are also constant during the entire test. On the other hand, the interior strain obtained by the FBG sensor shows a decreasing trend as in the case of the previous experiment. As explained earlier, due to the transverse cracks and other damage mechanisms, permanent deformations are accumulated in the composite material, which results in non-uniform strain/displacement fields on the entire specimen. Therefore, local strains at the center area of the specimen may vary significantly in comparison to the global behavior. The overall decrease of axial FBG strain in displacement-controlled fatigue test (which is about 800 με) is more noticeable when compared to the extensometer-controlled one. The displacement-controlled fatigue experiment leads to a larger drop in strain because the entire specimen length between the grip heads is taken into consideration as the gauge length. Therefore, the longer gauge length means that the applied strain is averaged over a larger length, thereby causing a more variable strain distribution on the entire specimen. The variation between the average strain and the strain at the exact FBG location (i.e., 1 mm long) is therefore more noticeable in the displacement-controlled fatigue experiment. The FBG strain has also three distinct phases, which correlate quite well with those observed in Figure 9a,b.

The force data in Figure 9a indicate very similar behavior in comparison to the previous experiment. Three distinct phases in the force versus cycle number plot are clearly visible. The Phase I behavior is particularly pronounced for this experiment, which means that the accumulated damage is considerably higher than the previous experiment. This situation is most likely related to the higher strain rate (i.e., 0.55 vs. 0.6) employed in this experiment. Figure 9b yields the variation of temperature for the specimen under fatigue loading. Here, the temperature of the specimen increases sharply due to the autogenous heating. The temperature attained at the end of the first thermal phase is noticeably higher than that for Specimen 1 due to the higher strain rate utilized in this specimen. The length of the first thermal phase complies with the first fatigue phase given in Figure 9a. Afterwards, the temperature declines and gradually levels off. This region is called the second thermal phase, which is in agreement with the second fatigue phase in Figure 9a. The decrease in temperature unlike Specimen 1 is owed to the rate of heat generation being smaller than the rate of heat removal to the environment given that the convective heat flux from the surface of the specimen increases with the rise in ΔT. At a later phase, there is a noticeable deviation in temperature from the linear region, such that the temperature drop is augmented, indicating the onset of the third thermal phase.

**Figure 10:**
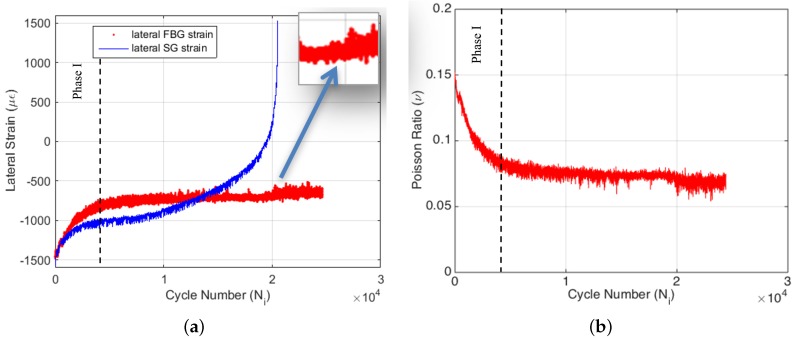
Lateral strain data (**a**) and Poisson’s ratio reduction (**b**) for Specimen 2.

In the previous experiment, it has been pointed out that the strain-gauges may not work properly for high strain ratio experiments. The displacement-controlled test also indicated that the cyclic axial strain is not measurable with strain-gauges for high strain ratios. On the other hand, the lateral strain data were recorded by the lateral part of the biaxial strain-gauge. However, as seen in Figure 10a, the lateral strain data deviate from the lateral FBG data around 13,000 cycles. After this point, the strain-gauge reads incorrect strain values, while the lateral FBG still works properly. Moreover, the lateral strain-gauge signal was lost sooner than the failure of the specimen. This also supports that the strain-gauges are unreliable and may show abnormal behavior during the cyclic fatigue experiments of high strain ratios. Meanwhile, the lateral FBG signal holds on very well until the failure. In this experiment, the Phase III behavior of the lateral strain is only slightly noticeable. Hence, the related segment is magnified and given as the inset in Figure 10a.

The calculated Poisson’s ratio reduction is given in Figure 10b. The initial value of Poisson’s ratio is identical to that of the first experiment since Poisson’s ratio is a material property and, hence, is independent of the control type of the experiment. However, the reduction trend is different from the first experiment. The Phase I and Phase II segments of reduction are perfectly visible in Figure 10b. Phase III behavior starts around 19,000 cycles and is weakly observable in lateral FBG strain data. Nevertheless, the reduction of Poisson’s ratio is again successfully monitored using biaxially-located FBG sensors during the entire fatigue life of Specimen 2. Accordingly, the application of biaxially-embedded FBG sensors in FRP composites is proven to be an excellent candidate for structural health monitoring purposes.

### 3.3. Low Strain Ratio Fatigue Test

**Figure 11:**
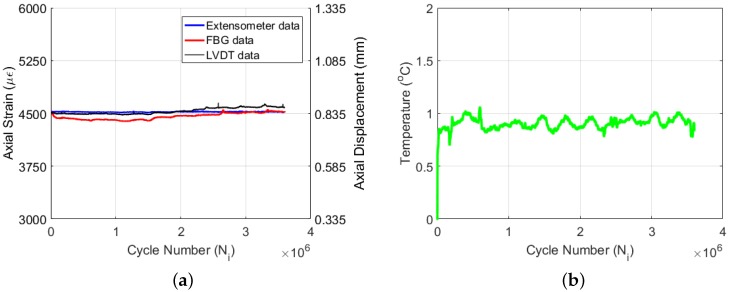
Force data (**a**) and temperature change (**b**) for Specimen 3.

To be able to illustrate the efficacy of the embedded biaxial FBG sensor for monitoring Poisson’s ratio reduction under a low amplitude high cycle fatigue test condition, we have conducted an extensometer-controlled constant tension-tension strain fatigue test on Specimen 3 at a strain ratio of (i.e., applied strain/ultimate strain) 0.27. More than 3.5 million cycles of fatigue data are collected, and the specimen did not fail under this test condition. Therefore, the data presented here do not indicate the full fatigue life of the tested material. Figure 11a yields the axial strain data acquired by the extensometer, FBG and LVDT sensors as a function of cycle number. Unlike the previous two experiments, the strain values of the extensometer, FBG and LVDT sensors do not deviate too much from each other, implying that damage in the composite specimen under such a small strain ratio is more limited. The overall decrease of axial FBG strain with respect to the extensometer strain is about 100 με. The recorded temperature variation data (i.e., ΔT) of Specimen 3 during the fatigue experiment are given in Figure 11b. The autogenous heating causes a sharp temperature increase at the beginning of the experiment; however, the amount of the increase is very limited (i.e., 0.8 °C) due to the low strain ratio of the experiment when compared to the previous ones. Following the initial increase, the temperature rises as high as 1 °C and then oscillates between 0.8 and 1.0 °C. The slow damage accumulation within the material keeps the rate of heat generation relatively small, which can be well balanced by the heat removal rate, whereby the temperature of the specimen does not change significantly and is almost constant throughout the experiment.

**Figure 12:**
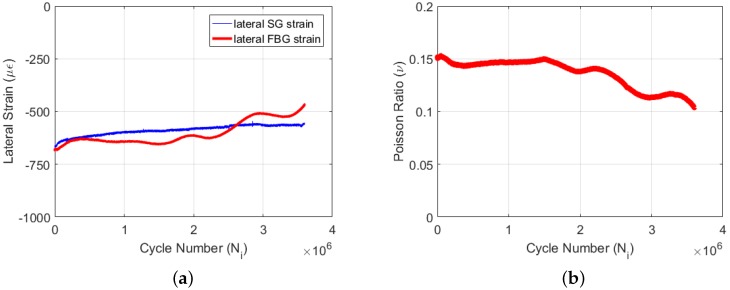
Lateral strain data (**a**) and Poisson’s ratio reduction (**b**) for Specimen 3.

The lateral strain data of both the FBG and strain-gauge sensors are given in Figure 12a. One can note that as the fatigue-induced damage accumulates within the specimen, the lateral FBG strain starts deviating from the strain of the lateral strain-gauge, since the damage affects the strain field around the embedded FBG sensor more so than the lateral strain values of the surface-mounted strain-gauge. The Poisson’s ratio reduction for Specimen 3 is given in Figure 12b. The starting value of Poisson’s ratio is in conformity with the previous experiment data. Since under a small strain ratio, the damage accumulation is expected to be a rather slow process, until 1.5 million cycles, Poisson’s ratio does not show significant reduction and, thereafter, declines in a nearly linear manner with a small amplitude and frequency oscillation during the whole experiment. Given that the collected data do not cover the entire fatigue life of the specimen, Figure 12b does not include the Phase III region of the fatigue experiment. However, this experiment renders the generality of the proposed methodology, the reliability of the biaxial FBG sensor for high cycle fatigue monitoring and the validity of the results, such that the Poisson’s ratio reduction can be effectively monitored by using a biaxially-embedded FBG sensor irrespective of the magnitude of the strain ratio.

## 4. Conclusions

Strain measurement has always been one of the most important practices for understanding structures’ response to mechanical effects. Biaxial strain measurement has many benefits, such as the determination of the Poisson’s ratio of materials, among others. For structures made of FRP composites, it is essential to be able to obtain continuous information from the material’s interior. In this context, the current study investigated the usage of embedded dual FBG sensors written on a single fiber optic cable to obtain biaxial strain data for monitoring the variation of Poisson’s ratio of composites, which can be used for determining the fatigue stages and, in turn, the structural health monitoring of composites. To this end, fatigue experiments were performed on specimens with dual FBG sensors under high and low strain ratios (i.e., 0.6, 0.55 and 0.27), and the FBG sensor-based biaxial strain data were successfully collected for each cycle throughout the tests. The reduction in Poisson’s ratio as a function of the fatigue cycle number was monitored successfully and in a repeatable manner by using embedded FBG sensors during the entire fatigue life of the structure and was shown to be well correlated with the fatigue stages based on the decrease in the applied fatigue force (equivalently stiff reduction in constant strain fatigue experiments) and the variation in both displacement and temperature. It was shown that surface-mounted strain-gauges are prone to breakdown under fatigue loading and, hence, cannot be used effectively throughout the fatigue experiment for monitoring the variation of Poisson’s ratio because of their poor high strain performance, thereby making the embedded dual FBG sensor an indispensable tool for Poisson’s ratio-based structural health monitoring.
